# Advancing the debate on hotel employees’ environmental psychology by promoting energy-saving behavior in a corporate social responsibility framework

**DOI:** 10.3389/fpsyg.2022.990922

**Published:** 2022-09-16

**Authors:** Long Yang, Jacob Cherian, Muhammad Safdar Sial, Sarminah Samad, Jongsik Yu, Youngbae Kim, Heesup Han

**Affiliations:** ^1^Zhengzhou Preschool Education College, Zhengzhou, China; ^2^College of Business, Abu Dhabi University, Abu Dhabi, United Arab Emirates; ^3^Department of Management Sciences, COMSATS University Islamabad (CUI), Islamabad, Pakistan; ^4^Department of Business Administration, College of Business and Administration, Princess Nourah bint Abdulrahman University, Riyadh, Saudi Arabia; ^5^College of Business Division of Tourism and Hotel Management, Cheongju University, Cheongju-si, Chungcheongbuk-do, South Korea; ^6^Division of Global Economics and Commerce, Cheongju University, Cheongju-si, Chungcheongbuk-do, South Korea; ^7^College of Hospitality and Tourism Management, Sejong University, Seoul, South Korea

**Keywords:** environmental psychology, sustainable individual behavior, CSR, social identity, hotel

## Abstract

Considering the vulnerable climatic conditions in most parts of the planet, a successful transition toward a carbon-free future is a critical challenge worldwide. In this respect, around 35% of the world’s total greenhouse gas emission (GHG) is associated with the power sector (especially electrical energy). To this end, a vast of electrical energy has been used by the people in buildings. Specifically, a significant amount of energy in buildings is used for heating, cooling, and ventilation. While the available literature highlights the importance of neat, clean, and green electrical energy for the decarbonization of society, a critical gap exists in such literature. That is, most of the literature under this stream deals with the supply side (production) of electrical energy, while the demand side (consumption at an individual level) was neglected. To bridge this critical knowledge gap, this study investigates how the CSR engagement of a hotel organization can promote the energy-related pro-environmental behavior (ERPEB) among the employees with the intervening effect of employees’ environmental commitment (EMEC) and Green intrinsic motivation (GRIM). Further, the conditional indirect role of altruistic values was also tested in this study. The data were collected from different hotel employees in Pakistan with the help of a self-administered questionnaire. We tested the hypothesized relationship through structural equation modeling (SEM). The results confirmed that CSR can be a potential motivator to impact the ERPEB of employees, while EMEC and GRIM mediated this relationship significantly. The findings of this study also confirmed the conditional indirect role of altruistic values. These findings offer various theoretical and practical contributions which are conversed in detail.

## Introduction

The rising phenomenon of climate change and other environmental issues have emerged as critical issues for many societies worldwide. Global warming, irregular weather cycles, poor air index, polluted water, rising floods, and droughts in various regions of the planet are some of the critical challenges of this age (Kong et al., 2021). Considering the hazards associated with climate change, researchers and practitioners have already indicated that every segment of an economy has to contribute if this world has to hope for a carbon-free future (Berger and Wyss, 2021). Recent climate data by the United Nations (UN) has already mentioned that to mitigate extreme climatic conditions to have a livable planet, the temperature increase at the global level needs to be restricted by 1.5°C above the pre-industrial era (United Nations, 2022). Nonetheless, the planet has already become warmer to almost 1.1°C compared to the 1800s. To make the matter worse, emissions in different regions of the world continue to increase. As per the Paris Agreement, worldwide emissions need to be mitigated by around 45% by the end of 2030 and should reach a net zero in 2050 (Paris Agreement, 2015).

Although the global community has taken several steps to mitigate the global carbon footprint, much work is still needed in this regard. According to a recent UN report, the energy sector is one of the largest carbon dioxide CO_2_ emitters. Especially, the electricity-related power sector is responsible for generating for generating around 35% of total greenhouse gas emissions (GHG; United Nations, 2018). In this respect, most of the electrical energy has been utilized by the people in the buildings (both in offices and homes). Indeed, Climate change data show that consumption at an individual level contributes to about 60% of the world’s GHG emissions (Ivanova et al., 2016). To deal with the environmental vulnerability, the concept of clean and green energies has been discussed in the recent literature to reduce the carbon footprint of the power sector (Chien et al., 2021; Zahan and Chuanmin, 2021; Akhtar et al., 2022). However, a careful review of the literature indicates the existence of a critical gap in the existing. That is, to date, bulk of the available literature has addressed the environmental concerns by dealing with the supply side of energy (the production side; Rastogi et al., 2018; Chien et al., 2021), whereas the demand side of energy (the consumption at an individual level) was less emphasized in the literature on this domain. We feel that advancing the debate on the energy-saving behavior of an individual is important, at least for two reasons. First, the available literature indicates that individuals have a critical role in the decarbonization of a society (Ivanova et al., 2016; Peng et al., 2022); therefore, it is worthwhile to investigate major factors that can promote the energy-saving behavior of the individuals. Second, from the perspective of electricity consumption, the environmental data suggest that most of the electrical energy in buildings has been consumed by individuals for cooling or heating purpose (Energy, 2015; Arif et al., 2021). Buttressing this debate, UN data on climate change show that overall emissions related to cooling and heating purposes in buildings, will likely to rise by 90% by 2050 if not managed on a war-like footing (United Nations, 2018). This again highlights the role of individuals in decarbonization.

In an organizational context, encouraging the sustainable behavior of individual employees is valuable (Kong et al., 2021; Murtaza et al., 2021). The literature on organizational management indicates that certain employee behavior is influenced by different organizations (Wiederhold and Martinez, 2018; Wang and Chen, 2019), and personal factors (Gifford and Nilsson, 2014; Nguyen et al., 2019). To this end, the role of corporate social responsibility (CSR) efforts of an organization was highlighted in the literature as an enabler in influencing different employee behaviors (Ahmad et al., 2022b,c). However, the potential role of CSR in influencing the energy-saving behavior, which is identified in the literature as energy-related pro-environmental behavior (ERPEB) of employees, remained an under-investigated terrain. Therefore, a critical objective of this study is to investigate the role of CSR in influencing the ERPEB of employees in an organizational context.

Similarly, at a personal level, literature on employee psychology highlights different personal factors that, in an organizational milieu, influence employee outcomes. In this regard, the literature on green employee behavior has indicated the mediating role of employees’ environmental commitment (EMEC; Afsar and Umrani, 2020; Sharma et al., 2021) and green intrinsic motivation (GRIM) in predicting the sustainable behavior of employees (Faraz et al., 2021). However, the literature under this stream too did not reveal the mediating potential of these two factors to promote ERPEB at the level of employees. Hence, another critical objective of this study is to investigate the mediating effects of EMEC and GRIM between CSR and ERPEB.

The role of personal values in behavior formation was mentioned in the literature (Osman-Gani et al., 2010; Eva et al., 2017). Precisely, from an environmental aspect, literature discusses the importance of altruistic values, which focus on the wellbeing of others, to influence the PEB of employees (Shao et al., 2021). Although the central character of values in influencing the behavior of employees was highlighted in the literature, it was also mentioned that values provide only a general guideline to influence human behavior (Zasuwa, 2016). This shows that values require a specific framework to influence a certain human behavior, implying that investigating their indirect role is more important than a direct impact. Therefore, this study proposes the conditional indirect effect of altruistic values between the mediated relationship of CSR and ERPEB through EMEC and GRIM.

All in all, the current study tends to advance the available literature by offering the following insights. First, it enriches the literature related to clean and green energies from a consumption aspect, whereas most of the prior literature has attempted to address the environmental issues by dealing with the supply side of energy (the production side; Rastogi et al., 2018; Chien et al., 2021). Second, this is one of the limited studies on environmental management from the energy-saving behavior perspective of employees in a hospitality context which was less emphasized earlier. Because the hospitality sector uses a sheer amount of electricity for heating, cooling, and lighting purposes (Natalie, 2019), it will be worthwhile to investigate the proposed relationships. Lastly, this study attempts to advance the existing literature by proposing a robust model to promote ERPEB of employees by simultaneously testing the mediating and moderating effects of EMEC, GRIM, and ALTV, which, according to our best knowledge, were not tested earlier.

Similarly, practically this study tends to help the hospitality sector in the following ways. Firstly, this study helps the hospitality sector to improve its environmental footprint by promoting the energy-related sustainable behavior of employees in a CSR framework. Especially from an electricity consumption perspective, this point is critical because a significant amount of electrical energy in Pakistan has been wasted by individuals (including employees) due to their inappropriate behavior toward electricity consumption. Therefore, it will be important to improve their energy-friendly behavior for a better and carbon-free future from a CSR perspective. Secondly, this study also tends to help Pakistan in dealing with electricity shortfall by improving the sustainable behavior of employees in a hospitality context. This is important to realize because evidence suggests that the hospitality sector uses a large amount of electricity for heating, cooling, and lighting purposes. Especially in Pakistan, almost 75% of electricity is used in buildings, among which around 40% of electricity is consumed for cooling, heating, or ventilation (Arif et al., 2021). Thus, by promoting ERPEB of employees, such a large electricity consumption may be significantly reduced.

## Literature

This study employs the theoretical lens of social identity theory (SIT) which Tajfel originally proposed in the 1970s (Tajfel, 1978) who contends that individual perceptions about personal identity are strongly influenced by others during a process of social interaction. In social psychology, this theory explains the interplay between an individual’s personal and social identities by specifying certain circumstances under which people associate or identify themselves with a certain social group (Harwood and Roy, 2005). Indeed, this theory originates from the belief that certain social groups are able to instill meaning in their group members in different social situations. This theory is regarded as an integrative theory because it tends to connect individual cognitive processes with behavioral motivation (Vinney, 2019). Specifically, this theory discusses the psychological processes of individuals through which they identify themselves with a social group by focusing on three social aspects: social categorization, comparison, and identification (Hogg, 2000). In this regard, social categorization is related to the likelihood of individuals perceive themselves and other people with respect to specific social categories. Similarly, social comparison relates to the process by which individuals determine the relative social value of a particular group. Lastly, social identification relates to the notion that individuals usually do not tend to evaluate a social situation as detached observers because their own sense (who they are and how they perceive others) implicate in the way they perceive a social group (Trepte, 2013). Hence, an individual’s social identity is the outcome of these three social processes.

Although the origin of this theory lies with the work of Tajfel, however in 1989, Ashforth and Mael (1989) were the persons who brought this theory to organizational sciences literature. They emphasized that in an organizational milieu, employees’ personal identity is influenced by different workplace factors, including organizational (social group) environment, culture, etc. Since its emergence into the organizational sciences literature, various scholars have used this theory to elucidate certain human behaviors in the workplace. In a CSR context, behavioral scientists have subscribed to this theory to explicate various employee outcomes (Hu et al., 2020; Sun et al., 2020; Deng et al., 2022). In relation to the current context, our argument here is that the social and ethical commitment of a corporation offers employees with a foundation to develop personal identification with an ethical enterprise. Hur et al. (2018) referred to this process as a “sense-making” process on the part of the workforce due to CSR. This sense-making process motivates employees to back their ethical firm to achieve different sustainability-related objectives in the larger interest of society, the biosphere, and future generations. Other scholars have also used this theory in different contexts to explain employee behaviors, especially their extra-role behaviors (like ERPEB) due to their social identification with a social group (the organization; Paruzel et al., 2021; Raza et al., 2021; Ahmad et al., 2022a). All in all, the employees’ perceptions regarding the ethical commitment of their organization encourage them to strongly identify themselves with the organization, and thus they willfully partake in different pro-social activities by acting pro-environmentally in the workplace.

The positive association between CSR and PEB of employees has been mentioned in the previous literature on employee psychology (Alzaidi and Iyanna, 2021; Murtaza et al., 2021). Yu et al. (2021) mentioned that the ethical orientation of a firm is evaluated positively by the employees which ultimately encourages them to act pro-socially. The other researchers have also mentioned that the CSR commitment of a firm is well placed in the literature on employees’ extra-role behavior (like PEB; Vlachos et al., 2014). From an energy-saving perspective, some recent scholars have argued that a socially responsible organization takes various steps to mitigate its environmental footprint by taking different energy-saving measures (for example, installing solar energy instruments or replacing the existing machinery with energy-friendly equipment; Fu et al., 2022b). Employees observe such moves of their organization keenly and in return, are expected to respond positively by changing their attitude and behavior toward energy consumption (Fu et al., 2022b). Also, with respect to the social identity theory, we expect that workers feel a strong social bond with their employer due to its socially responsible engagement. This social bond is something that motivates the employees to strongly identify themselves with a social group (the ethical organization). Ultimately, the social identification with an ethical organization motivates employees in a manner that enhances the social image (the CSR related) of their organization which eventually urges them to act pro-environmentally, especially by using less amount of electricity. Therefore,

*Hypothesis 1*: The CSR engagement of an organization is expected to positively influence ERPEB.

Literature regards commitment as a psychological mind state that explains the degree to which an individual employee shows commitment to an organization (Meyer and Herscovitch, 2001). It was also emphasized in the literature that employee commitment is a critical psychological factor in predicting employee behaviors (Rita et al., 2018; Massoudi et al., 2020). Nonetheless, the bulk of the prior literature on employee commitment discusses it from a general aspect. In this respect, some recent researchers have investigated the role of employee commitment in guiding a certain behavior of employees (Pham et al., 2019; Cop et al., 2020). With respect to the context of this study, we describe EMEC by using the definition by Cantor et al. (2012), who defined EMEC as “a person’s feelings of internal obligation to preserve the nature and biosphere through his or her actions.”

Recent CSR literature shows that EMEC can significantly induce when they serve a socially responsible organization (De Silva and De Silva Lokuwaduge, 2021; Loor-Zambrano et al., 2022). Specifically, Afsar and Umrani (2020) believed that perceptions of employees with respect to the CSR commitment of a firm could be linked to their environmental commitment. Advancing the debate on employee organizational psychology in relation to CSR and social identity theory, we are in agreement with past researchers who believed that CSR engagement of an organization motivates employees to show a higher commitment level to support the sustainability objectives (Unsworth et al., 2021; Fu et al., 2022b). In a nutshell, an employee with an enhanced level of commitment is motivated to solve different challenges that their social group faces (Kim et al., 2017), and because such commitment takes shape from the CSR commitment of a firm, we expect CSR influences EMEC which then mediate between CSR and ERPEB. Thus:

*Hypothesis 2*: There is a positive association between the CSR engagement of an organization and EMEC.

*Hypothesis 3*: EMEC mediates the relationship between CSR and ERPEB.

The general literature on the intrinsic motivation of employees mentions that as a psychological factor, an employee’s intrinsic motivation encourages him or her to be engaged in different tasks that are interesting and offer an inner level of satisfaction (Minh-Duc and Huu-Lam, 2019). Specifically, an internally motivated employee shows an enhanced level of motivation to achieve some objectives due to inner satisfaction and not for any extrinsic reward (salary or other financial benefits, for example). This study uses the definition of intrinsic motivation by Deci and Ryan (2013) and modifies it in the current context as “GRIM of employees is a psychological process which internally motivates employees to complete a task with respect to environmental consideration.” The past CSR literature has already documented that as employees’ CSR perceptions improve, their intrinsic motivation level also improves in an organizational context (Hur et al., 2018; Kunz, 2020). Particularly, the literature related to positive employee psychology indicates that employees positively identify themselves with an ethical organization, ultimately improving their intrinsic motivation (Hao et al., 2018). Moreover, Glavas (2016) indicated that an organization’s ethical commitment is something that infuses the feelings of pride among employees to be the workers of a socially responsible organization. The mediating effect of intrinsic motivation to influence different employee outcomes has been established in prior literature on employee psychology (Shareef and Atan, 2019; Danish et al., 2020). Even from an environmental perspective, Faraz et al. (2021), showed that GRIM is a significant mediator in predicting the PEB of employees. Specifically, for a long-term and sustained PEB, the literature highlights the need to internalize it by linking it with the intrinsic level of employee motivation (Ryan and Deci, 2000). The other authors like Taufik et al. (2015) also provided an intrinsic motivational foundation to induce the PEB of employees by stressing that individuals are motivated to act pro-environmentally beyond the monetary rewards. Referring to the seminal work by Andreoni (1990), it is argued that when individuals decide to act pro-socially, they think it is right to do due to their intrinsic motivation. Further, Van Der Linden (2015) extended the debate on the relationship between intrinsic motivation and PEB by stating that compared to extrinsic motivation (which is short-lived), an individual with intrinsic motivation can act pro-environmentally for a long period of time. Moreover, a growing body of knowledge has already acknowledged the seminal role of intrinsic motivation in spurring the PEB of employees (De Groot and Steg, 2010; Graves et al., 2019). Even in the literature related to environmental management, the recent study by Faraz et al. (2021) verifies the mediating effect of GRIM to predict the PEB of employees. To conclude, as CSR influences the intrinsic motivation of employees positively, and employees with an enhanced level of intrinsic motivation show a better engagement to act pro-environmentally, we propose:

*Hypothesis 4*: CSR engagement of an organization induces GRIM of employees.

*Hypothesis 5*: GRIM of employees mediates between CSR and ERPEB.

Past literature indicates that the personal values of an individual have a seminal role in guiding human behavior (Pirson et al., 2017; Kruse et al., 2019). For instance, it was specified in the literature that personal values for collectivism (altruistic values also focus on the collective benefit of all) could significantly influence the behavioral intentions of people (Hueso et al., 2020). Similarly, from a consumer perspective, the literature verifies the role of personal values in influencing the purchase likelihood behavior of consumers (Frank et al., 2015). From an environmental perspective, Barbarossa et al. (2017) mentioned that people’s environmental values encourage them to engage in activities that limit an individual’s environmental footprint by adopting energy-friendly technologies, for instance, electronic vehicles. Reflecting the role of personal values at an employee level, Zientara and Zamojska (2018) found that the environmental values of an employee boost the commitment level of an employee to act pro-environmentally. A recent survey conducted by Sabokro et al. (2021) revealed that the personal values of an individual employee for green environmentalism promote the sustainable behavior of employees in a workplace.

**Figure 1:**
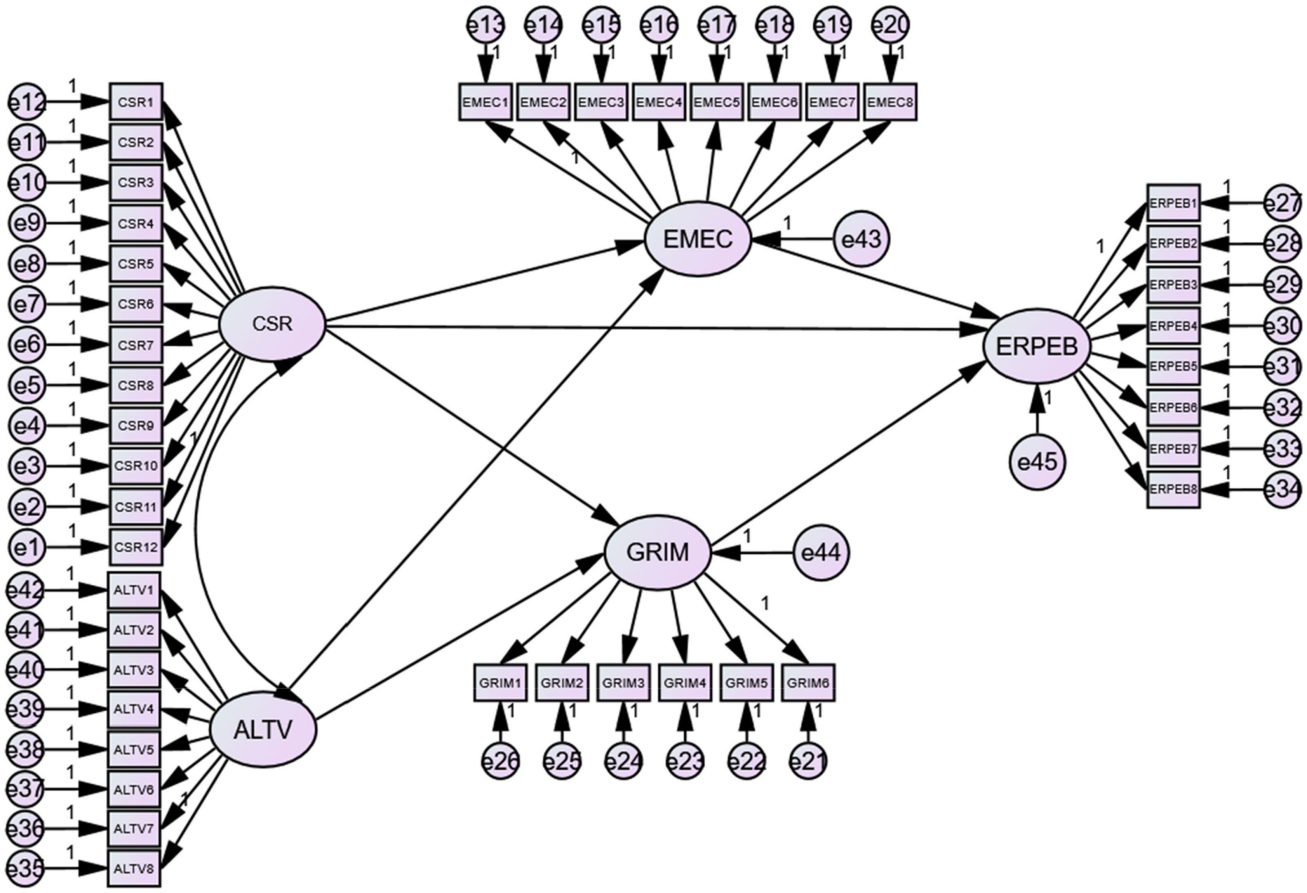
The hypothesized structural model: CSR, Corporate Social Responsibility (X); EMEC, Employee Environmental Commitment (M1); GRIM, Green Intrinsic Motivation (M2); ERPEB, Energy Related Pro-Environmental Behavior (Y); ALTV, Altruistic Values (W); CSR_x_ALTV, Interaction term.

The role of altruistic values in predicting the PEB of employees has well-known roots in the existing academic debate on organizational sciences and environmental management literature (van Riper et al., 2020; Xu et al., 2022c). Even from an energy-related standpoint, the existing literature has recognized the critical part of altruistic values in predicting the ERPEB of employees. Though personal values influence individual behavior, however, the value framework of an individual only provides a general guideline for behavior formation. This highlights that the personal values of an individual require a specific context (the ethical context of an organization in the current study) to form a specific behavior (ERPEB). This is one of the reasons that behavioral scientists have tested the indirect impact of values to predict a certain behavior. For instance, Al-Ghazali and Afsar (2021) have recently verified green values have a conditional indirect effect on fostering green creativity behavior of employees. Similarly, the study by Deng et al. (2022) emphasized the conditional indirect role of altruistic values in influencing the PEB of employees due to CSR. Moreover, the impact of altruistic values improves the environmental commitment of individuals was also reported by extant researchers (Perkins and Brown, 2012; Rahman and Reynolds, 2016). Similarly, we are in agreement with Steg (2016) who has argued that individuals are intrinsically motivated to act pro-socially when they contain robust altruistic values. The author further stressed that such biospheric values require contextual support (CSR in the current case) to strongly motivate individuals to act pro-socially. In a nutshell, as the focus of the altruistic values of an individual is to work for the welfare of others, the same is the central focus of an organization under a CSR philosophy (Figure 1). Therefore, with reference to the above discussion, we propose the following hypotheses:

*Hypothesis 6*: Altruistic values are expected to moderate the mediated relationship between CSR and ERPEB *via* EMEC.

*Hypothesis 7*: Altruistic values are expected to moderate the mediated relationship between CSR and ERPEB *via* GRIM.

## Materials and methods

### Data collection process

Worldwide, the tourism and hospitality industry has been growing. Recent UN data show that around 1 billion tourists visit some international destination annually (United Nations World Tourism Organization, 2014). In addition, the tourism and hospitality sector showed a growth rate of almost 4% before the outspread of the Coronavirus in late 2019 (United Nations World Tourism Organization, 2019). In combine, it was specified in a recent report that tourism and hospitality along with its allied sectors contributed almost 5 trillion US dollars to the global economy in 2020 (before the spread of Covid-19).

Specifically, the hospitality segment grew at a fast pace in the recent past throughout the globe as the number of international arrivals was more than 200% compared to 2016 (increased from 600 million to 1.4 billion; Insights, 2020). Although the economic evidence of tourism and hospitality is strong, however, there is another perspective associated with this sector. That is, at a global level, this sector is identified as a dirty segment with respect to the environment because, in combine, the tourism sector contributes almost 8% to the world’s total GHG emissions (Lenzen et al., 2018; Koçak et al., 2020). The hotel segment alone contributes around 1% of total GHG at a global level.

For this study, we have targeted the hotel segment of Pakistan, which is a developing country in South Asia. The South Asian nation has recently emerged as an important destination for tourists. Recent evidence suggests that the hospitality sector in Pakistan will grow further in the coming years. However, like other regions in the world, Pakistan’s tourism and hospitality sector is not as green as it should be. In fact, the environmental issues of Pakistan have increased during the recent two decades. Environmental data suggest that every segment of the economy in the country needs to take strict measures for decarbonization. Hotels in Pakistan use a sheer amount of electricity for heating, cooling, and lighting purposes (Natalie, 2019). Therefore, reducing the carbon print of this sector is worthwhile by promoting the ERPEB of employees.

Particularly, the hotel sector in Pakistan constitutes a mixed structure that includes different national and international hotels. We selected Lahore city of Pakistan to collect the data from the hotel employees for this research. This city was considered due for the following reasons. First, Lahore is a mega city that is famous for tourism and hospitality among national and international tourists. This is why many national and international hotel chains operate in this city. Second, from an environmental perspective, Lahore is known for its outsized carbon emissions. Indeed, the city has the highest rank among the world’s most polluted cities (IQAir, 2021). The public health of the masses in this city is at risk due to poor environmental quality and requires serious attention for corrective actions.

We, in this respect, identified that different upscale hotels have designated CSR programs and can serve the purpose of data collection for this survey. Thus, we contacted different hotels to allow us to contact their employees for the sake of the data collection. Those hotels which responded positively (Six upscale hotels) were then approached for the data collection. Employees from different departments and designations were included in this survey.

Further, we followed the major ethical guidelines given in the Helsinki Declaration as specified by the extant researchers (Alam et al., 2021; Ullah et al., 2021). To collect the data, we employed a three-wave data collection strategy. Each wave was administered at an interval of 2 weeks. The data collection activity was completed from January to March 2022.

### Instrument

The data collection instrument of this study was an adapted questionnaire that was designed on a five-point Likert scale. The items to measure the variables in this study were taken from different published and reliable sources. The initial version of the questionnaire was presented to the experts (academia and hotel industry) to assess the suitability of the adapted items to serve the purpose of this study (Adnan et al., 2021; Awan et al., 2021). Originally, the questionnaire was divided into two sections, which included the socio-demographic information of the respondents and five-point items related to ratings. The questionnaire was self-administered in nature. Lastly, the printed version of the questionnaire was provided to each respondent in this study.

### Sample size and data cleaning

To decide on the minimum number of samples, we used the *a priori* sample size calculator which was designed by Dniel (2010). This calculator is specially designed for structural equation modeling, which is one of its major strengths of this calculator. Specifically, this calculator uses some input information to decide a minimum number of samples for a specific study. Recent researchers have also mentioned the suitability of this calculator for any structural model. In this respect, there were six latent variables (unobserved), and 42 observed variables (items). When we provided this input information to the calculator, it indicated that the minimum sample for this study should be 400. To keep the number of responses above this number, we initially distributed 600 surveys among the hotel employees. We received a total of 437 filled questionnaires; however, some of the questionnaires were not included in the final dataset because they contained partially filled information, or some of the responses were identified as outliers. After data cleaning and removal of outliers, we included 411 responses into the final dataset of this study. For further detail, we refer to Table 1. We used the famous Mahalanobis technique to decide whether an observation should be identified as an outlier. The output of the Mahalanobis test showed that 08 items were identified as outliers which were removed from the dataset Table 2.

**Table 1:** Data cleaning, outliers, and response rate.

	**Distributed**	**Returned**	**Unreturned**	**Unusable**	**Outliers**	**Final**
	600	437	113	26	08	411
Percentage	–	72.83	27.17	05.95	01.83	68.50

**Table 2:** Observations identified as outliers.

Observation number	Mahalanobis d-squared	*p*1	*p*2
142	14.391	0.002	0.037
79	13.166	0.008	0.016
44	12.410	0.022	0.142
303	09.683	0.016	0.138
156	09.255	0.030	0.079
90	08.927	0.033	0.092
28	08.713	0.039	0.098
194	08.156	0.045	0.062

The sample profile is given in Table 3. This is in line with prior researchers (Rasool et al., 2022).

**Table 3:** Sample profile.

**Demographic**	**Frequency (*n* = 411)**	**Percentage (%)**
Gender		
Male	305	74.21
Female	106	25.79
Age		
18–25	48	11.68
26–30	92	22.38
31–35	87	21.17
36–40	67	16.31
41–45	59	14.35
Above 45	58	14.11
Experience		
1–4	67	16.31
5–7	172	41.85
8–10	98	23.84
Above 10	74	18.00
Total	**411**	**100.00**

Bold value represents total number of respondent and total of percentage.

### Measures

There variable CSR was the predictor in this study which was measured by adapting 12 items from the scale developed by Turker (2009) which is one of the most famous scales to measure CSR perception of employees (illustrated items included: “This hotel implements special programs to minimize its negative impact on the natural environment’ and ‘This hotel encourages its employees to participate to the voluntary activities’”). Energy-related pro-environmental behavior was the criterion variable in this survey for which we adapted 8-items from the study of Blok et al. (2015). The original scale includes various items to measure different PEB of individuals; however, in line with the context of this study, we used 8-electricity related items (heating or cooling, lighting, and computer use). Illustrated items from this scale include “when I leave my office for a considerable period of time, and there is no one else, I switch off the lights and ‘I switch off my computer/notebook when I go home.”

There were two intervening variables in this survey (EMEC, and GRIM). To measure EMEC, we used the 8-items from the study by Raineri and Paillé (2016) which included the illustrated items “the environmental concern of my hotel means a lot to me” and “I feel a sense of duty to support the environmental efforts of my hotel.” Similarly, to measure GRIM, we adapted six items from the study by Li et al. (2020), which is originally based on the study by Blok et al. (2015).

Lastly, to measure altruistic values—ALTV, we used the scale developed by Schwartz (1992), which consisted of eight items (illustrated item included: ‘As a guiding principle in my life, I consider working for the welfare of others). We used a five-point importance scale ranging from not important (1) to extremely important (5) for this scale.

### Common latent factor

To detect the potential issue of common method variance (CMV), we performed a common latent factor (CLF) test in AMOS. For this purpose, we constructed two measurement models, one was the original hypothesized model with no CLF, and the other was constructed by including a CLF. The latent factor was allowed to produce a direct effect on all of the observed items. Both models were assessed to see the degree of variability (>0.2) in factor loadings of each item. The comparison of the standardized factor loadings revealed that both models did not vary significantly, (all loadings showed a variance <0.2). This indicates that a CLF was not able to produce a significant variance in the measurement model, implying that a CMV issue was not critical to deal with in this study.

## Results

### Reliability and validity

Confirming the convergent validity and reliability was the first stage in the data analysis phase. In this regard, we checked and confirmed the convergent validity of each variable by using the standardized factor loadings of each item of a variable. Through these standardized factor loading, we calculated the average variance extracted (AVE) of each variable by using the following formula (Equation 1).


(1) $$\mathrm{AVE}=\frac{\sum_{\dot{i}=1}^{k}\lambda_{i}^{2}}{\sum_{\dot{i}=1}^{k}\lambda_{i}^{2}+\sum_{i=1}^{k}.\mathrm{var}\left(\varepsilon i\right)}$$

Based on the above formula, the AVEs for all variables were beyond 0.5 (for further detail please see Table 4). Specifically, the AVEs values ranged from 0.519 (GRIM) to 0.563 (ERPEB). The established role here is that if the value of AVE for a variable is above 0.5, it is assumed that the variable has good convergent validity. Thus, the convergent validity of all variables was significant. Next, we calculated the composite reliability (CR) of all five variables by using the below formula (Equation 2).


(2) $$\mathrm{Composite reliability}=\left(\left(\sum \lambda i\right)2\right)/\left(\sum \lambda i\right)2+\sum \mathrm{var}\left(\varepsilon i\right)$$

**Table 4:** Validity and reliability.

	***λ***	***λ*** ^**2**^	**E-Variance**
CSR			
	0.809	0.654	0.346
AVE = 0.560	0.722	0.521	0.479
CR = 0.938	0.744	0.554	0.446
	0.708	0.501	0.499
	0.703	0.494	0.506
	0.806	0.650	0.350
	0.719	0.517	0.483
	0.836	0.699	0.301
	0.769	0.591	0.409
	0.733	0.537	0.463
	0.714	0.510	0.490
	0.700	0.490	0.510
ERPEB			
AVE = 0.563	0.738	0.545	0.455
CR = 0.911	0.712	0.507	0.493
	0.734	0.539	0.461
	0.702	0.493	0.507
	0.718	0.516	0.484
	0.823	0.677	0.323
	0.818	0.669	0.331
	0.746	0.557	0.443
EMEC			
	0.758	0.575	0.425
AVE = 0.542	0.749	0.561	0.439
CR = 0.904	0.701	0.491	0.509
	0.706	0.498	0.502
	0.752	0.566	0.434
	0.816	0.666	0.334
	0.702	0.493	0.507
	0.700	0.490	0.510
GRIM			
	0.728	0.530	0.470
AVE = 0.519	0.731	0.534	0.466
CR = 0.866	0.750	0.563	0.438
	0.700	0.490	0.510
	0.702	0.493	0.507
	0.709	0.503	0.497
ALTV			
	0.705	0.497	0.503
AVE = 0.539	0.709	0.503	0.497
CR = 0.903	0.722	0.521	0.479
	0.706	0.498	0.502
	0.742	0.551	0.449
	0.767	0.588	0.412
	0.783	0.613	0.387
	0.733	0.537	0.463

*λ*, item loadings; CR, composite reliability; ∑λ^2^, sum of square of item loadings; E-Variance, error variance.

It was observed that the CR values in all cases were beyond the normal value of 0.7 which shows that all CR values were significant (CSR = 0.938, ERPEB = 0.911, EMEC = 0.904, GRIM = 0.866, ALTV = 0.903). Thus, the initial analysis revealed that the validity and reliability of each variable were significant.

### Model fitness

After the establishment of validity and reliability, we next assessed whether the theoretical model of this study fits well with the data compared to other alternate models. For this purpose, we constructed four measurement models (Table 5) with different combinations and compositions. In this regard, model 1 was a five-factor model (original hypothesized) whereas all other models were alternate models. To decide on the superiority or inferiority of different measurement models, we compared different model fit indices (especially NFI and CFI) of each model. It was realized that the model fit indices in the case of model 1 were superior compared to all other cases (NFI = 0.958, CFI = 0.956). This shows that model 1 fits the data more appropriately compared to all other models. Further, we also compared other model fit values, for example, *χ*^2^*/df* and RMSEA for all measurement models. It was realized that the model 1 had the most appropriate values (*χ*^2^*/df* = 2.369 and RMSEA = 0.052). Moreover, Δ*χ*^2^*/df* comparison revealed that the variance ranged between 0.687 and 3.759.

**Table 5:** Model fit comparison, alternate vs. hypothesized models.

Model	Composition	*χ* ^2^ */df*	Δ*χ*^2^*/df -*	NFI	CFI	RMSEA
		(<3)	–	(>0.9)	(>0.9)	(<0.08)
1	(hypothesized) CSR, ERPEB, EMEC, GRIM, ALTV	2.369	**_**	0.958	0.956	0.052
2	(3-factor) CSR + EMEC, ERPEB+GRIM, ALTV	6.128	3.759	0.711	0.700	0.089
3	(2-factor) CSR + ALTV+EMEC, GRIM+ERPEB	7.233	1.105	0.602	0.599	0.161
4	(1-factor) CSR+ ERPEB+EMEC+ GRIM+ ALTV	7.920	0.687	0.528	0.521	0.212

### Correlations

Next, we continued with the data analysis phase and performed a correlational analysis. For this reason, correlation (*r*) values between different pairs of variables were assessed. It was realized that all values were positive and significant. The highest value was found in the case of GRIM-ALTV = 0.533, which was significant. All other comparisons also verified that *r* values were positive and significant. These values provide initial support to the theoretical statements of hypotheses. On a further note, no pair of variables showed a critical value of *r* (beyond 0.8), indicating that multicollinearity was not evident in any case of this dataset. We also assessed our variables for discriminant validity. While a convergent validity analysis is important to see whether the items of a variable are converging on to it, the case of divergent validity is important to realize whether the items of one variable are different from other variables. In this regard, we took the square root of AVE for all variables separately (the bold diagonal values in Table 6). These values were then compared to the values of *r.* It was observed that *r* values were inferior in every case, establishing that the divergent validity was significant.

**Table 6:** Correlations and discriminant validity.

Construct	CSR	ERPEB	EMEC	GRIM	ALTV	Mean	SD
CSR	**0.748**	0.419	0.386	0.400	0.526	3.061	0.610
ERPEB		**0.750**	0.289	0.428	0.511	2.896	0.577
EMEC			**0.737**	0.382	0.462	2.882	0.580
GRIM				**0.720**	0.533	3.290	0.580
ALTV					**0.734**	2.972	0.556

Bold diagonal values represent discriminant validity of a variable.

### Hypotheses evaluation

To test the hypotheses in this survey, we used SEM analysis in AMOS software (Deng et al., 2022; Fu et al., 2022a; Ahmad et al., 2022b,c). Further, we also took into consideration the guidelines given in the PROCESS Macro. This Macro enabled us to calculate the conditional indirect effect of ALTV at three different levels (at mean, above 1 SD of the mean, and below 1 SD of mean). Specifically, the guidelines of Model-7 were observed. Further, a user-defined syntax in AMOS was employed to calculate different equations given in Model 7 of PROCESS Macro. We also converted CSR and ALTV to mean-centered prior to proceeding with this analysis. Additionally, an interaction term (CSR_X_ALTV) was also generated for this analysis. Bootstrapping option in AMOS was used to see the significance of mediation and moderation effects. We have reported all the results related to hypotheses analysis in Table 6. This SEM analysis revealed that CSR positively influenced ERPEB, EMEC, and GRIM of employees (0.4128, 0.3925, 0.3996). These values were significant because *p* < 0.05 in all cases; moreover, no value of confidence interval contained a zero point (0.398–0.563, 0.287–0.496, 0.242–0.506). This provides statistical support to accept H1, H2, and H4.

To decide on mediation effects, we observed the bootstrapping results (a 5,000 bootstrapping sample was used). It was observed that both EMEC and GRIM significantly mediated between CSR and ERPEB (CSR ➔ EMEC ➔ ERPEB = 0.1986; CSR ➔ GRIM ➔ ERPEB = 0.2365, *p* < 0.05 in all cases with non-zero confidence interval values). These results were more than enough to arrive at a conclusion that H3 and H5 were supported by statistical evidence.

Lastly, to test the conditional indirect effect of ALTV between CSR and EMEC and between CSR and GRIM, three points were taken into the consideration (at mean value of ALTV, above 1SD of mean, and below 1SD of mean). The highest values achieved in this respect are reported in Table 7 for the convenience of readers. The statistical evidence support that ALTV provides a buffering effect between the mediated relationship of CSR and ERPEB through EMEC and GRIM. This gives support to accepting the theoretical statements of H6, and H7.

**Table 7:** Hypotheses testing.

Hypotheses	Estimates (SE)	*t*/*z*	Value of *p*	CI
(CSR ➔ ERPEB) (CSR ➔ EMEC) (CSR ➔ GRIM)	0.4128 (0.0396) 0.3925 (0.0402) 0.3996 (0.0398)	10.4242 09.7636 10.0402	0.000 0.000 0.000	0.398, 0.563 0.287, 0.496 0.242, 0.506
Mediating effects				
(CSR ➔ EMEC ➔ ERPEB)	0.1986 (0.0258)	07.6976	0.000	0.119, 0.328
(CSR ➔ GRIM ➔ ERPEB)	0.2365 (0.0283)	08.3568	0.003	0.178, 0.374
Conditional indirect effect When EMEC is mediator	0.2240 (0.0269)	08.3271	0.000	0.169, 0.388
Conditional indirect effect When GRIM is mediator	0.2726 (0.0258)	10.5658	0.007	0.220, 0.384

CI = 95% confidence interval with lower and upper limits.

## Discussion

The statistical findings of this study confirmed that the CSR engagement of a socially responsible hotel organization could promote PEB among the employees, especially from an energy-saving perspective. An ethical hotel organization pursues different decarbonization strategies, such as installing solar energy equipment as an alternative to fossil fuel energy. Employees observe such moves of their organization keenly and, in return, are expected to respond positively by changing their attitude and behavior toward energy consumption. On a further note, referring to the theory of social identity and the process of sense-making, the employees of an ethical hotel are expected to put forth extra efforts to maintain the ethical identity of their social group (the organization). Thus, the process of sense-making urges them to support the sustainability objectives of their organization, especially toward energy saving. All this process encourages them to adopt a sustainable behavior toward energy saving and consumption. This finding is in line with the findings of some recent researchers who argued that different organizational factors like leadership and CSR could motivate employees to act pro-socially, especially from an energy perspective (Peng et al., 2022; Xu et al., 2022a). Especially in a recent study Xu et al. (2022b) mentioned that the CSR and leadership style of a hotel organization significantly predicted ERPEB. Similarly, a related outcome was derived by Xu et al. (2022a) in a healthcare context. Therefore, CSR activities of a hotel organization can have a definite role in predicting the PEB of employees, especially from an energy-saving and consumption perspective, as indicated by the statistical results of this study.

Our results further confirmed that EMEC significantly mediates between CSR and ERPEB in a hospitality context. In this regard, we argue that employee commitment especially to preserve the environment, improves with an improvement in their CSR perceptions regarding their ethical hotel. Specifically, when employees see that their organization is taking different steps toward energy efficiency under its CSR plan, they also become more self-responsible and show a higher level of environmental commitment. This finding is in line with Afsar and Umrani (2020), who verified that the environmental commitment of employees improves as an outcome of their CSR perceptions regarding their organization. Employees with a greater level of environmental commitment show extra concern for preserving the environment and biosphere and hence they act pro-socially in the workplace (Unsworth et al., 2021; Fu et al., 2022b). Hence, the mediating effect of EMEC was confirmed in this study. More specifically, when an organization considers environmental improvement under its CSR strategy, employees’ environmental concern also increases, and they are expected to treat their ethical organization as their own place. In other words, in response to CSR, employees start to own their ethical organization and support it in every aspect that may reduce the environmental footprint of their organization.

The statistical results of this study also confirmed that GRIM mediates between CSR and ERPEB. Our argument here is that the CSR perceptions of employees improve, and their level of intrinsic motivation also improves. Particularly, in line with the literature on positive employee psychology, we expect that employees positively identify themselves with an ethical organization which ultimately improves their intrinsic motivation. This finding also receives support from extant researchers (Hao et al., 2018). Additionally, an intrinsically motivated employee shows an enhanced level of motivation to achieve some objectives due to inner satisfaction and not for any extrinsic reward (salary or other financial benefits, for example). Especially from long energy-related sustainable behavior, it is important for an organization to internalize it by linking it with the employees’ intrinsic motivation. Referring to the work of Taufik et al. (2015) an intrinsic motivational base to induce the PEB of employees is long-lasting compared to external motivation which is short-lived. Our results are in line with the past literature that different organizational factors (CSR in the current case) positively influence GRIM, which then mediates between CSR and PEB (Ahmed et al., 2021). Additionally, employees with a higher level of intrinsic motivation put forth efforts to achieve their goals due to their inner feelings and satisfaction and not for external rewards. When looked from this perspective, we expect that intrinsically motivated employees will show a better motivation to be engaged in different energy-saving and consumption behaviors because such energy related behavior will satisfy their inner-self.

Lastly, our results verified that ALTV moderates the mediated relationships between CSR and ERPEB *via* EMEC and GRIM. The available literature on personal values has already mentioned the conditional indirect role of ALTV in forming a certain behavior of an individual (Zhou et al., 2018), including the PEB of employees (Deng et al., 2022). Our argument here is that as the central focus of ALTV is to prefer the wellbeing of others over personal interests; therefore, employees with high ALTV orientation are expected to reduce their energy consumption on their part. Further, there is value congruence between CSR and ALTV because both focus on the welfare of others. This value congruence motivates employees to a further level to show an enhanced level of environmental concern (Xu et al., 2022a). Specifically, when combined, CSR, ALTV, GRIM, and EMEC of employees positively influence their energy-related behavior by taking different initiatives on their part that could reduce the environmental footprint of a hotel organization significantly.

On a further level, the environmental values of an employee boost his or her commitment level to act pro-environmentally Sabokro et al. (2021). Similarly, referring to the work of Steg (2016), we argue that individuals are intrinsically motivated to act pro-environmentally when they have strong altruistic values. Moreover, such biospheric values require contextual support (CSR in the current case) to strongly motivate individuals to act pro-socially. All in all, the results of this study confirmed that in the presence of ALTV, there is a buffering effect between the mediated relationship of CSR and ERPEB through EMEC and GRIM.

### Theoretical contribution

Theoretically, this study advances the debate on organizational sciences and environmental management from an employee psychology perspective by promoting their energy-saving behavior in a CSR framework. In this regard, as we specified at the onset of this manuscript that the literature related to clean and green energies has attempted to address the environmental issues by dealing with the supply side of energy (the production side; Rastogi et al., 2018; Chien et al., 2021), whereas, the demand side of energy (the consumption at an individual level) was less emphasized in the literature on this domain. Considering the critical role of an individual in decarbonization, it was worthwhile to bridge this knowledge gap. Another important theoretical contribution of this study is that this is one of the limited studies on environmental management from the energy-saving behavior perspective of employees. Although the tourism and hospitality sector is notorious for its large carbon footprint at a global level, shockingly less literature discusses the climate change issue related to this sector from an employee perspective. Moreover, as the hospitality sector uses a sheer amount of electricity for heating, cooling, and lighting purposes (Natalie, 2019), it was worthwhile to bridge this knowledge gap by reducing the carbon print of this sector by promoting the ERPEB of employees. Lastly, this study advances the existing literature on employee psychology and organizational sciences by proposing a robust model to promote ERPEB of employees by simultaneously testing the mediating and moderating effects of EMEC, GRIM, and ALTV, which according to our best knowledge were not tested earlier. The potential strength of the proposed model of this study is that it considers organizational factors (like CSR), personal factors (EMEC and GRIM), and personal values (ALTV) in a unified model to explain ERPEB more robustly.

### Practical contribution

This study significantly helps to hospitality sector of Pakistan by providing the following practical insights. In the first place, this study helps the management of the hospitality sector to improve its environmental footprint by promoting the energy-related sustainable behavior of employees. For this, the hospitality management is required to carefully plan its CSR strategies, especially from an energy efficiency perspective, because employees’ energy-saving behavior is determined, at least in part, by the CSR activities of an ethical hotel organization. Especially from an electricity consumption perspective, this implication has special relevance to the hospitality sector, which is already known for its large environmental footprint because almost 25% of electrical energy in Pakistan has been wasted by individuals (including employees) due to their inappropriate behavior toward electricity consumption. Therefore, it is important to improve their energy-friendly behavior for a better and carbon-free future.

In the second place, not only do the results of this study provide practical insights into the hospitality sector of Pakistan from an environmental perspective, the findings of this study are equally important from an economic perspective too. Undoubtedly, enhancing economic efficiency is the ultimate objective of every enterprise, irrespective of the field and size of industry. In this respect, when employees show a caring attitude and behavior toward consumption and preservation of electrical energy, it reduces the carbon footprint of a hotel on one side, it also helps to improve the economic efficiency because by reducing the consumption of electricity, a hotel organization can save economic cost which ultimately improves its economic return.

In the third place, this study also helps Pakistan in dealing with electricity shortfall by improving the sustainable behavior of employees. Recent evidence suggests that the hospitality sector uses a large amount of electricity for heating, cooling, and lighting purposes which can be reduced by careful energy efficiency behavior (Patton, 2020). Moreover, considering that almost 75% of electrical energy in the country has been consumed by buildings, among which around 40% is associated with climate control (heating, cooling, and ventilation), it is worthwhile to help the country in dealing with the electricity shortfall by improving energy consumption behavior at an individual level. In this respect, we suggest that the CSR plan of a hotel organization should be closely aligned with the energy efficiency perspective.

### Limitations and possible future directions

This study faces some potential issues which may be realized as limitations. The first limitation of this study rests with the geographical consideration, as the current study collected the data only from Lahore. Though considering the large number of hotels that operate in Lahore and considering the poor environmental condition in this city, it was worthwhile to collect the data from hotel employees in this city. However, we still suggest including more cities in order to have better generalizability. Another potential issue rests with the nature of the data. The current survey was conducted by following a cross-sectional survey design in which the information was only collected at a specific point in time. Although cross-sectional surveys are very common in behavioral studies, establishing causal relationships under a cross-sectional survey method is difficult. Therefore, we suggest employing a longitudinal data design in future studies. A non-probability sampling method was another potential limitation of this survey. Given that due to different policy and safety issues, most hotels were not agreed to share with us any list of employees, which could serve as a sampling frame to apply a probability sampling, we were unable to introduce any probability sampling technique. There is not any doubt in believing that probability sampling is regarded as superior compared to non-probability sampling. Therefore, if possible, we suggest future studies should use a probability sampling (for example, random sampling) method. Moreover, as the perspective of managers is very important to foster EMEC and ERPEB in an organization, we suggest considering this perspective in future studies. Lastly, as indicated by the prior researchers, the potential role of digital transition in the context of energy consumption by employees may also be incorporated in future studies (Centobelli et al., 2020, 2021; Nwaila et al., 2022).

## Conclusion

This study verifies that the CSR activities of a hotel organization can influence the sustainable behavior of employees, especially from an energy-saving perspective which is of seminal importance. On a further note, this study advances the debate on clean and green energy by indicating that only focusing on the supply side of energy is insufficient, and for a better and sustainable future, it is equally important to improve the consumption side of energy. For this, a well-planned CSR strategy could be a way forward because CSR strategies of an organization from an energy-related perspective can affect the energy consumption behavior of employees in an organizational milieu. Moreover, considering the complex nature of human behavior, it is important to consider the seminal role of personal factors and personality characteristics in shaping human behavior. To this end, our results have highlighted the role of EMEC, GRIM, and ALTV for which the CSR engagement of a hotel organization is equally important. Additionally, we suggest the management of a hotel organization to improve EMEC, GRIM, and ALTV of employees by providing them with special training programs under the umbrella of CSR because when such training and developmental plans are aligned with the main CSR plan, the employees will better realize the importance of their individual roles for a carbon-free future. We also suggest the management of a hotel organization to reconsider the employee hiring procedure and criteria. In this respect, a screening procedure should be developed that can identify individuals with high welfare values with a better environmental commitment to improving the biosphere with their active participation. All in all, by improving ERPEB as an outcome of CSR, the country can hope for a successful transition toward a carbon-free economy.

## Data availability statement

The raw data supporting the conclusions of this article will be made available by the authors, without undue reservation.

## Author contributions

All authors listed have made a substantial, direct, and intellectual contribution to the work and approved it for publication.

## Funding

This work was funded by Princess Nourah bint Abdulrahman University Researchers Supporting Project number (PNURSP2022R4) Princess Nourah bint Abdulrahman University, Riyadh, Saudi Arabia.

## Conflict of interest

The authors declare that the research was conducted in the absence of any commercial or financial relationships that could be construed as a potential conflict of interest.

## Publisher’s note

All claims expressed in this article are solely those of the authors and do not necessarily represent those of their affiliated organizations, or those of the publisher, the editors and the reviewers. Any product that may be evaluated in this article, or claim that may be made by its manufacturer, is not guaranteed or endorsed by the publisher.
