# Supplementary material for: Advancing the debate on hotel employees’ environmental psychology by promoting energy-saving behavior in a corporate social responsibility framework
Source: Front Psychol. 2022 Sep 16;13:990922. doi: 10.3389/fpsyg.2022.990922 (PMC9524265; doi:10.3389/fpsyg.2022.990922)
Supplement: Supplementary file 1 [file Data_Sheet_1.pdf]

Appendix-A: Items used in this study

CSR

This hotel participates in the activities which aim to protect and improve the quality of the natural environment

This hotel makes investment to create a better life for the future generation

This hotel implements special programs to minimize its negative impact on the natural environment

This hotel targets sustainable growth, which considers to the future generations

This hotel supports non-governmental organizations working in the problematic areas

This hotel contributes to the campaigns and projects that promote the well-being of the society

This hotel encourages its employees to participate in the voluntary activities

This hotel policies encourage the employees to develop their skills and careers

The management of this hotel is primarily concerned with employees' needs and wants

This hotel implements flexible policies to provide good work and life balance for its employees

This hotel's decisions related to the employees are usually fair

This hotel supports employees who want to acquire additional education

ERPEB

I check whether thermostats are set correctly in my office

I wear more/less clothes instead of putting the heating/air conditioning on.

I make sure that heating/airconditioning is off or reduced outside working hours

I reduce heating/air conditioning in unused rooms.

I switch off electricity devices (computer/notebook, etc.),when I leave my office for a considerable period

I switch off electricity devices (computer/notebook, etc.) when I go home

I switch on the lights when I come to the office in the morning and switch them off while leaving

When I leave my office for a considerable period of time, and there is no one else, I switch off electricity devices

EMEC

I really care about the environmental concern of my hotel

I would feel guilty about not supporting the environmental efforts of my hotel

The environmental concern of my hotel means a lot to me

I feel a sense of duty to support the environmental efforts of my hotel

I really feel as if my hotel's environmental problems are my own

I feel personally attached to the environmental concern of my hotel

I feel an obligation to support the environmental efforts of my hotel

I strongly value the environmental efforts of my hotel

GRIM

I am strongly motivated by the recognition which I can earn from my organization for environmental tasks

I often think about rewards, salary, or promotions for my environmental tasks

I want other people to find out how good I really can be at my environmental

tasks
I have to feel that I am earning something for my environmental tasks
I’m concerned about how other people are going to react to my environmental ideas

ALTV

As a guiding principle in my life, I consider working for the welfare of others
As a guiding principle in my life, I consider working for the unity with nature
As a guiding principle in my life, I consider working for the prevention of pollution
As a guiding principle in my life, I consider working to protect the environment
As a guiding principle in my life, I consider working to respect the Earth
As a guiding principle in my life, I consider working for the social justice
As a guiding principle in my life, I consider working for a world at peace
As a guiding principle in my life, I consider working for equality
